# A Review of Microfiber-Based Temperature Sensors

**DOI:** 10.3390/s18020461

**Published:** 2018-02-04

**Authors:** Wanvisa Talataisong, Rand Ismaeel, Gilberto Brambilla

**Affiliations:** Optoelectronics Research Centre, University of Southampton, Southampton SO17 1BJ, UK; rmni1g10@orc.soton.ac.uk (R.I.); gb2@orc.soton.ac.uk (G.B.)

**Keywords:** microfibers, nanofibers, fiber resonator, fiber coupler, temperature sensor

## Abstract

Optical microfiber-based temperature sensors have been proposed for many applications in a variety of industrial uses, including biomedical, geological, automotive, and defense applications. This increasing demand for these micrometric devices is attributed to their large dynamic range, high sensitivity, fast-response, compactness and robustness. Additionally, they can perform in-situ measurements remotely and in harsh environments. This paper presents an overview of optical microfibers, with a focus on their applications in temperature sensing. This review broadly divides microfiber-based temperature sensors into two categories: resonant and non-resonant microfiber sensors. While the former includes microfiber loop, knot and coil resonators, the latter comprises sensors based on functionally coated/doped microfibers, microfiber couplers, optical gratings and interferometers. In the conclusions, a summary of reported performances is presented.

## 1. Introduction

**Figure 1:**
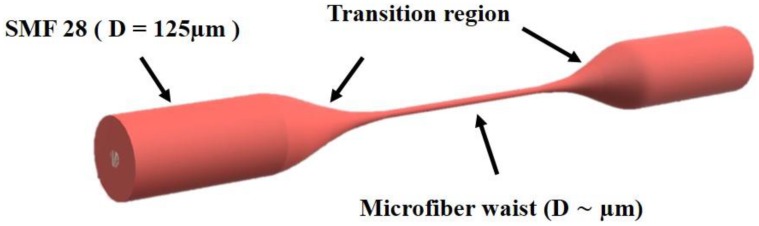
Schematic of an optical MF. A uniform waist region (center) is connected to single mode optical fiber pigtails (SMF 28) by conical transition regions.

Microfibers (MFs) are unique devices formed by heating and stretching standard optical fibers to submicron dimensions [1]. The geometry of a MF (Figure 1) consists of a thin uniform waist connected from each side to a slowly varying transition region [2]. These MFs, which are sometimes referred to as nanowires [3], combine two elegant features of sub-wavelength dimensions simultaneously with low operation losses. These features have promoted MFs to be the ideal building block for a variety of components within the ever-growing field of nanotechnology. Ultra-fast response time, large evanescent field, compactness and tailored modal area are added bonuses for MFs to be used efficiently in sensing application [4]. A large number of research papers has therefore investigated the optimum design and shape of these MFs to tailor their output according to the required application. 

## 2. Microfiber Manufacture

MF fabrication is a relatively simple and low-cost process. Low loss, sub-micron diameter MFs were originally fabricated using a two-step technique [5], where the optical fiber is firstly drawn to a micron size using a flame and the resulting taper is then drawn down to submicron dimensions by pulling the taper around a heated 80 μm sapphire rod. This method allowed the fabrication of a taper with ~440 nm radius and a 0.3 dB/m loss at the telecom wavelength *λ* = 1.55 μm. Lower losses, on the order of 0.001 dB/mm at *λ* ~ 1.55 μm, were obtained by using the so-called flame brushing technique.

**Figure 2:**
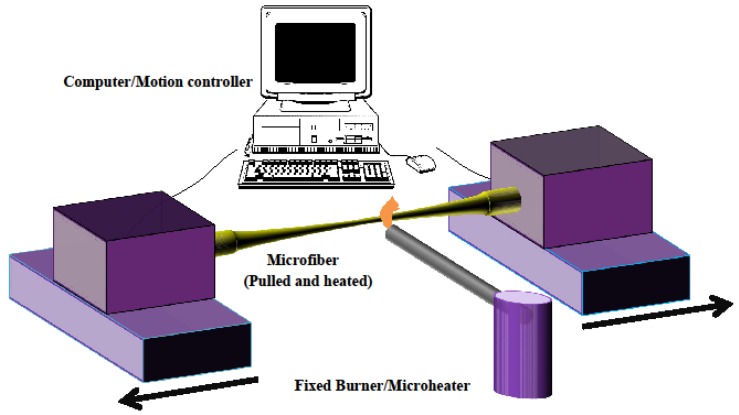
The flame brushing technique: a flame travels under an optical fiber clamped at its extremity onto two computer-controlled stages which are moving apart.

The standard setup frequently used for fabricating MFs is shown in Figure 2. The fiber is pulled by two translation stages that have submicron precision while a small region of the fiber is heated by either a flame (flame brushing technique) or by an electric microheater (modified flame brushing technique) [2].

**Figure 3:**
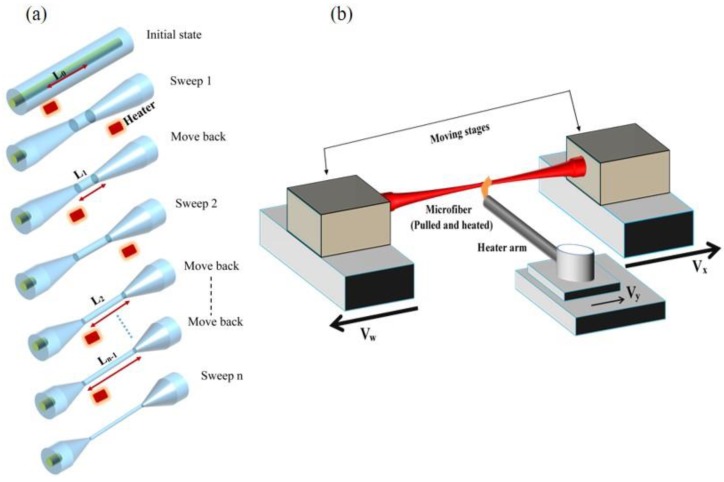
Schematic of (**a**) taper profile evolution using the multi-sweep tapering method; (**b**) Experimental implementation of the single-sweep tapering method.

Manufacturing MF and MF sensors is expected to move soon to the next level, with recent techniques allowing for the precise monitoring of the MF diameter during fabrication [6,7,8]. A ‘multi-sweep’ (Figure 3) tapering method (based on the flame brushing technique) was demonstrated to optimize the ratio of feed and draw velocities at each tapering sweep, yielding a precise shaping of the MF [7]. In this technique, the ratio of the feed and draw velocities can change within each tapering sweep, enabling the manufacture of more precise and complex MFs. A quantitative analysis showed that the mismatch error in this technique decreases by increasing the number of tapering sweeps and shortening the length of the hot-zone formed by the heater.

Real time MF waist diameter monitoring with ultrahigh accuracy and precision was achieved during pulling by checking the excited high-order modes in a SMF28e fiber coupled to a CW laser operating at *λ* ~ 785 nm. During the fiber-pulling process, the cut-off of high-order modes contributed to sudden transmission intensity drops. Precise information regarding the final fiber diameter could be obtained by accurately measuring the time interval between two drops, and hence determining the time required to stop the pulling process based on a target diameter [9]. This technique allows for an accurate diameter control within 5 nm.

MF manufacturing using CO_2_ lasers was proposed and relied on using a camera operating in the infrared to check the fiber alignment and the laser power profile. Monitoring the fiber transmission spectrum during tapering allows to ensure that the taper will have the required spectral properties, which can be optimized for specific sensing applications [10]. Fiber Bragg gratings (FBGs) were used during pulling to monitor/control the MF strain, and therefore its profile. 

Manufacturing arbitrary waist profiles was demonstrated using the modified flame-brush technique by approximating the taper diameter function to any monotonic function of the fiber length while combining a superposition of step-tapers [11]. A theoretical approach to the manufacture of complex-shaped tapered fibers was based on controlling the motion of the heat source relative to the fiber, as well as its temperature distribution [12].

## 3. MF Sensor Properties

One of the most interesting MF properties for sensing applications is the tailorability of its transmission to the required applications. It is possible to optimize the sensor sensitivity, detection limit and dynamic range by simply optimizing the MF design. 

**Figure 4:**
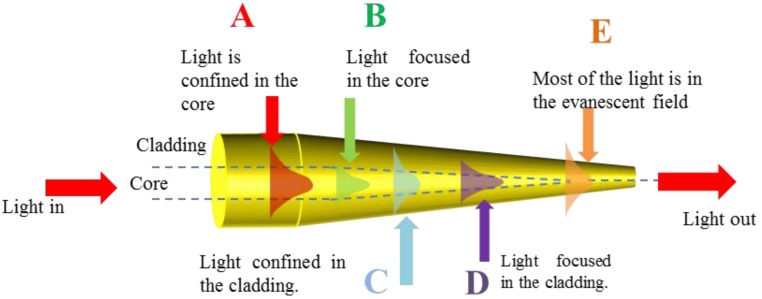
Modes evolution along the length of the tapered optical fiber.

Figure 4 shows the mode evolution along a MF transition region. While in untapered fiber (**A**), modes are confined by the core/cladding interface, in the decreasing section (**B**) light is initially focused in the decreasing size core until core guidance becomes weak and the mode expands (point **C**) and the mode is guided by the cladding/air interface: here the mode diameter is maximum. The mode is then confined by the cladding/air interface and reveals the maximum confinement (**D**), which makes this area interesting for nonlinear applications and corresponds to MF diameters around 1 μm for silica in air. For smaller diameters (**E**), most of the light propagates in the evanescent field outside the air/silica interface: this region is particularly important for sensing applications as it allows maximum interaction with the surrounding.

### 3.1. Low Loss

Losses in MF are attractively low, which is an important feature for sensing applications that require appropriate signal to noise (S/N) ratios. The best reported optical losses at *λ* ~ 1.55 μm for silica MFs are of the order of 10^−3^ dB/mm [13,14,15]. Low loss in MFs are mainly related to the MF surface roughness and adiabatic profile. A MF is considered adiabatic when two modes with propagation constants $\beta_{1}$ and $\beta_{2}$ propagate unperturbed and therefore do not exchange power. 

**Figure 5:**
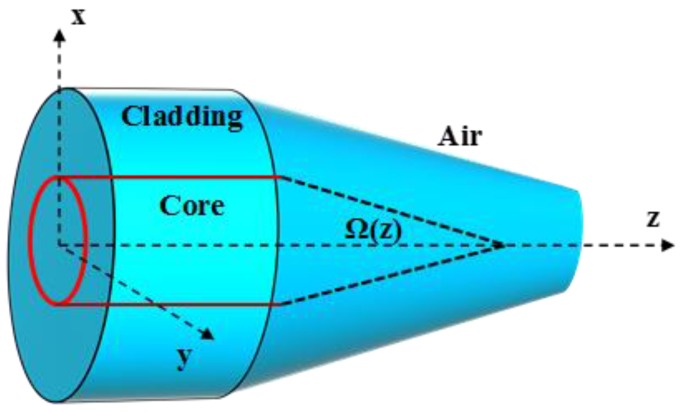
Schematic diagram illustrating the local tapering angle Ω in the transition region.

The adiabatic angle $\bar{\Omega }$ in the transition region (Figure 5) of radius *r* can be written as a function of the beat length $z_{b}$, [4,16,17]:

(1) $$\bar{\Omega }=\frac{r}{z_{b}}=\frac{r\left(\beta_{1}-\beta_{2}\right)}{2\pi }$$

An adiabatic, lossless MF requires that Ω < $\bar{\Omega }$, thus that the fiber diameter changes smoothly ensuring a small transmission loss and intermodal coupling. According to Landau-Dykhne formula described in [18,19], optical losses in an ideal MF will experience an exponentially dependency on the MF diameter, therefore, MFs with diameters smaller than *λ*/10 will practically not be guided. Losses in MF, however, can also increase over time [11], with narrower waist MFs degrading faster than thicker ones [20,21]. Such time-dependent losses can be partially recovered by embedding MFs in low-refractive-index materials such as fluoro-chlorinated polymers, aerogel and resins [22,23,24].

### 3.2. Large Evanescent Field

**Figure 6:**
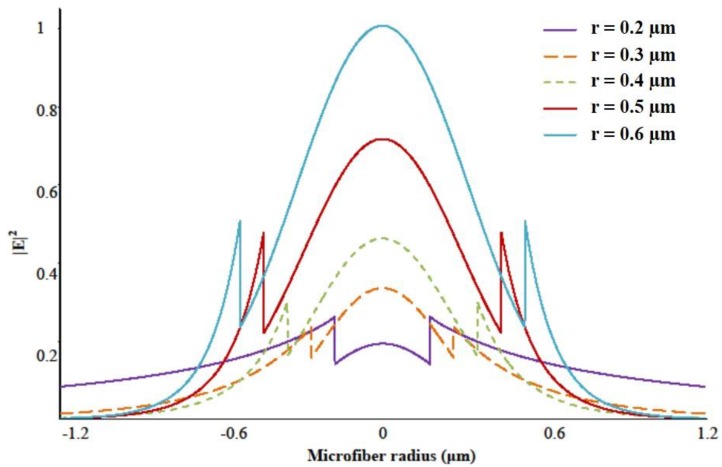
Mode field intensity profile for the HE_11_ mode at various MF radii *r*. The field discontinuity is presented as spikes at the silica-air interface.

Evanescent field is the fractional power propagating outside the MF physical boundary (Figure 6). It is an important property for designing sensors due to strong overlap, and thus interaction, of the MF evanescent field with the surrounding environment. 

As the MF diameter decreases, the guiding becomes weaker and the mode expands until it is confined by the cladding/air interface. As mentioned earlier, modes are tightly confined in the ~1 μm silica MF in air, but for extremely small diameters (*D* ~ 0.5 μm at *λ* ~ 1.55 μm) most of weakly guided modes propagate in the evanescent field outside the air/silica interface. 

**Figure 7:**
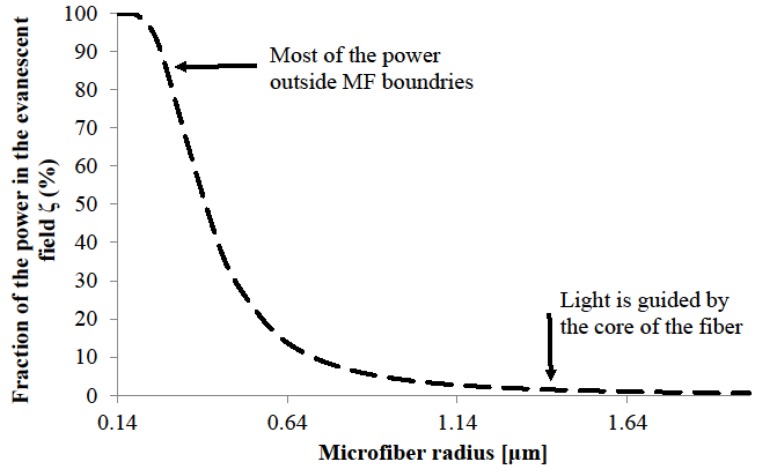
Fraction of the power in the evanescent field against MF radius, for a Silica MF in air at *λ* = 1.55 μm.

MF sensors are often coated with special compounds that have the required properties for specific applications. For example, bio-detectors based on MF are frequently coated with functionalizing materials (polymers, resins) which can selectively bind chemicals. This will induce an enhancement in the sensor specificity and sensitivity to select substances. Figure 7 show the dependency of the field distribution on the MF size. 

The amount of power propagating in the evanescent field ζ can be defined as ζ=1−η where η is the fraction of the power inside the core. 

### 3.3. Confinement

**Figure 8:**
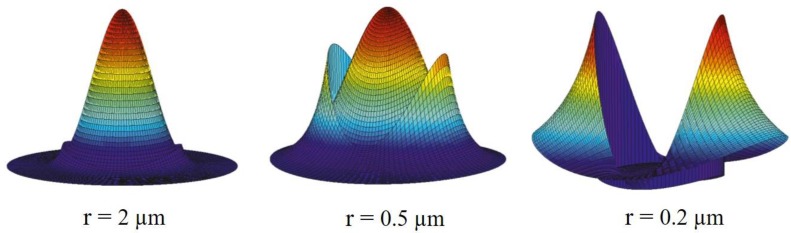
Field intensity distribution of silica MFs in air with radii of (**a**) 1 μm, (**b**) 0.5 μm and (**c**) 0.2 μm.

Due to the small area in the MF waist region, the propagating mode in such a region can be extremely confined. This feature was investigated in the manufacture of MF tip sensors for high compactness and sensitivity. Light, when confined in these sub-wavelength tips, has higher intensity than the original guiding fiber core itself. Figure 8 shows the mode confinement evolution as the diameter of a silica MF in air decreased from 4 to 0.4 μm. At MF diameters ~1 μm the beam spot-size ω is close to the diffraction limited value of *ω*_0_ ~ *λ*/2*nc*, where *n* is the silica refractive index and *c* the light speed in vacuum.

**Figure 9:**
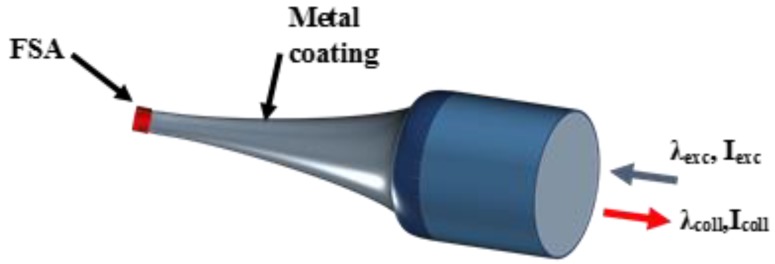
Schematic of a functionalised sensor based on light confinement.

The MF capability to confine light in small waist regions, together with an extremely small end-face cross section, have attracted extreme interest for bio-detection, intracellular sensing and plasmonic environment detectors as shown in Figure 9 [25]. 

### 3.4. Mechanical Strength

Despite MFs’ small size, they show extremely high strength. MFs manufactured using the heat and pull technique with diameters below 400 nm, recorded strengths values larger than 10 GPa [26,27]. However, different manufacturing techniques, like the self-modulated taper drawing technique, showed lower strength values. This was later attributed to the molecular-level smoothness of the MF surface, which can be easily degraded by defects created during manufacture [27].

### 3.5. Flexibility

Due to the high MF numerical aperture, it is possible to configure them into devices (like resonators) containing sharp bends and turns with negligible bending loss. These losses, however, become significant when the device size is decreased to few microns. Bending loss is an important design feature when manufacturing devices exploiting the evanescent field of the propagation mode, as this requires small *V* numbers and the bend loss coefficient $\alpha_{bend}$ has an exponential dependence on both the bending radius ρ and *V*:

(2) $$\alpha_{bend}=\frac{U^{2}}{2VWK_{1}(W)}\left(\frac{\pi }{rp}\right)\exp\left[-\frac{4W}{3V}\left(1-\frac{n_{MF}^{2}}{n_{surr}^{2}}\right)\frac{\rho }{r}\right]$$

ρ is the bending radius, $n_{MF}$ and $n_{surr}$ are the refractive indices of MF and of the surrounding medium, and *U* and *V* are the transverse wavenumbers, and *K*_1_ is the modified Bessel function of the second kind [28]. 

## 4. Sensor Parameters

Sensors performance is usually quantified by several parameters, which include: sensitivity, resolution, detection limit, response time, repeatability and operating range.

- *Sensitivity (S)* represents the change in the parameter used for detection as a result of a change in the parameter being monitored. For temperature sensing, this will either be in units (a) nm/°C when monitoring the shift in wavelength, or (b) dB/°C when monitoring the change in the power.
- *Resolution (R)* is the smallest detectable change in the parameter used for detection. The resolution is related to the precision with which the measurement is made and hence is usually affected by the specifications of the detection system.
- *Detection limit (DL)* represents the minimum quantity change in the parameter to monitor that can be detected by the sensor. It is related to *S* and *R* by:
  (3) $$DL=\frac{R}{S}$$
- *Response time (*τ*)* is typically the time required to rise to 90% of the final value, measured from the onset of the step input change in the parameter to monitor. It is frequently measured in s.
- *Dynamic range (DR)* is the range of values of the parameter under examination, which can be measured by the sensor.
- *Repeatability* is the variation in the detection parameter for measurements taken on the same sample under the same experimental conditions. It is an indicator of the agreement between two different measurements carried out on the same sample.

In the next sections MF temperature sensors will be grouped in two broad groups: non-resonant and resonant sensors. 

## 5. Nonresonant Micro/Nanofiber Temperature Sensors 

Nonresonant MF Temperature sensors are of great interest due to their compact size, high temperature sensing, high temperature sensitivity, and fast response. There are many structures used to fabricate nonresonant MFs, including functionalized MFs, MF couplers, MF gratings and MF interferometers. 

### 5.1. Functionally Micro/Nanofibers Surface

Glass MFs directly drawn from undoped materials are passive structures, thus functional activation is desired for temperature sensing applications due to the low glass thermo-optic coefficient. For glass MFs, one of the most convenient approaches is coating the MF with a functional film while maintaining its waveguiding capability. 

**Figure 10:**
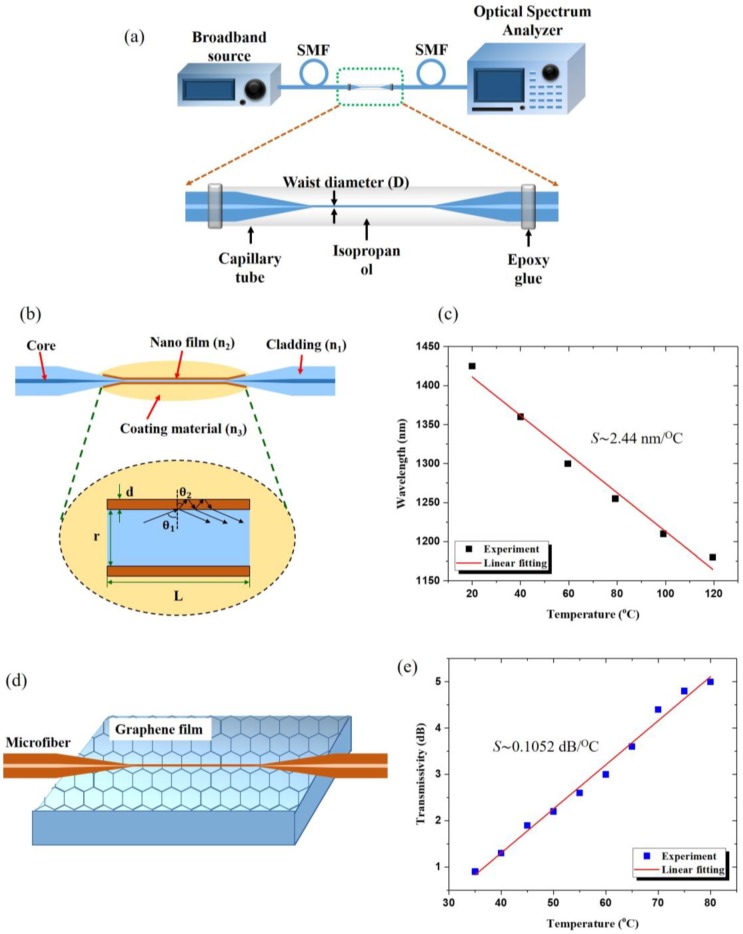
(**a**) Schematic diagram of isopropanol-sealed capillary. (**b**) Schematic diagram of the MF coated with the high refractive index Al_2_O_3_ nanofilm. (**c**) Dependence of the resonant wavelength shift of metal nanofilm coated MF for increasing temperatures. (**d**) Schematic diagram of graphene assisted MF (GAMF). (**e**) Transmissivity change of the GAMF for increasing temperatures.

In 2004, a temperature sensor based on MF for measuring liquids exploited a silica MF coated with a thermochromic film [29]. The sensing mechanism in this work relied on the temperature-dependent spectral absorbance of a thermochromic material (Iophine). Detection relied on the measurement of the transmitted power change and provided a sensitivity of 0.043 dB/°C at the fiber diameter of 35 μm. In 2013, a 7.2 μm diameter MF was inserted into a capillary filled with isopropanol (IPA) (Figure 10a), [30] and a temperature change from 20 °C to 50 °C produced a change in refractive index of the IPA surrounding the MF from 1.3766 to 1.3631, resulting in a change in the effective refractive index of light guided by MF, thus a shift of the output wavelength. The sensor revealed an ultrahigh temperature sensitivity of *S* ~ −3.88 nm/°C. In 2015 [31], a 10 μm diameter silica nonadiabatic MF was packed in Polydimethylsiloxane (PDMS) and used in a modal interferometer. The interference of HE_11_ and HE_12_ modes affected the MF transmission spectra showing a periodic behavior strongly dependent on the external refractive index. Temperature induces a change of the PDMS refractive index due to its high thermo-optic coefficient (−4.5 × 10^−^^4^/°C) [32] and by monitoring the wavelength shift with temperature, a temperature sensitivity of *S* ~ 3.102 nm/°C was achieved when the temperature was varied from 20 °C to 48 °C.

High temperature sensing has been demonstrated [33] by depositing Al_2_O_3_ nanofilms on 1.25 μm diameter MFs by using atomic layer deposition (ALD) technology [34]. The Al_2_O_3_-deposited MF was then coated with a temperature-sensitive silicone gel. Since the nanofilm has higher refractive index than the silica fiber taper, the light cannot be confined by total internal reflection. The nanofilm acts as a Fabry-Perot cavity (Figure 10b), therefore, the transmission spectrum presents a periodic interference pattern. The total wavelength shift of this sensor is 244.2 nm from 20 °C to 120 °C, resulting in the average temperature sensitivity of *S* ~ 2.44 nm/°C. 

The combination of the high thermal conductivity of graphene and the large evanescent field of MF was exploited to fabricate an efficient graphene-assisted MF (GAMF) thermometer [35]. The 1.984 μm waist diameter MF was placed above the graphene film coated on top of a low refractive index substrate (MgF_2_) to decrease the transmission loss of MF (Figure 10c). Due to the electrostatic force, the MF easily attached to the graphene leading to an interaction between the evanescent field of MF and graphene. Changes in the MF transmitted power associated to the effective refractive index change were ascribed to the variation of conductivity of graphene with applied temperature, resulting in a high sensitivity of *S* ~ 0.1052 dB/°C in the temperature range 30 °C to 80 °C. 

### 5.2. Microfiber Coupler 

**Figure 11:**
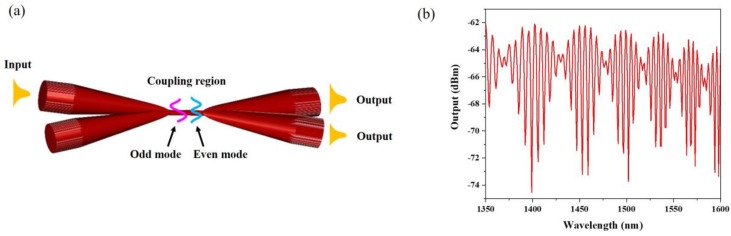
(**a**) Schematic of a MF coupler: two supermodes are excited at the coupler waist region. (**b**) An output spectrum from the MF coupler.

A second class of MF thermometers rely on MF couplers (MFC), which typically exhibit two input (P_1_ and P_2_) and two output ports (P_3_ and P_4_) and it is formed (Figure 11a) by combining together and fusing [36,37], etching [38,39,40] or polishing [41,42] two optical fibers. As a result, part or all power can be transferred between closely placed waveguides and when light is launched into one of the input ports, both even and odd supermodes are simultaneously excited in the coupling region. The wavelength dependent beating of supermodes leads to a continuous change in the power distribution along the coupler cross section, which results in a different power splitting at the output ports for different optical paths (Figure 11b).

The MFC coupling equations can be categorized into two regimes depending on the size of waist diameter and the degree of fusion. In the weakly fused coupler, the system is approximated as two touching cylindrical waveguides and the coupling coefficient for the *X* polarization (*C_x_*) and *Y* polarization (*C_y_*) are given by [43]:

(4) $$C_{x}+C_{y}=\frac{2^{7/2}{\left(n_{1}^{2}-n_{0}^{2}\right)}^{1/2}U_{\infty }^{2}}{n_{1}a\left(\sqrt{\pi }\right)V^{5/2}}$$

(5) $$C_{x}-C_{y}=\frac{2^{5/2}{\left(n_{1}^{2}-n_{0}^{2}\right)}^{1/2}U_{\infty }^{2}}{n_{1}^{3}a\left(\sqrt{\pi }\right)V^{7/2}}$$

In the strongly fused coupler, the two fiber cores are brought close to each other and the coupler cross section here has either a dumbbell, elliptical or circular profile. and *C_x_* and *C_y_* become [43]:

(6) $$C_{x}+C_{y}=\frac{3\pi \lambda }{32n_{1}a^{2}}\left[\frac{1}{{\left(1+\frac{1}{V}\right)}^{2}}+\frac{1}{{\left(1+\frac{n_{0}^{2}}{n_{1}^{2}}\cdot \frac{1}{V}\right)}^{2}}\right]$$

(7) $$C_{x}-C_{y}=\frac{3\pi \lambda }{16n_{1}a^{2}}\cdot \frac{1}{V}\left(1-\frac{n_{0}^{2}}{n_{1}^{2}}\right)$$

where *a* is the fiber diameter at the coupler waist region, *U*_∞_ = 2.405 and $V=\left[\left(2\pi a\right)/\lambda \right]{\left(n_{1}^{2}-n_{0}^{2}\right)}^{1/2}$ and *n*_1_ and *n*_0_ are the refractive indices of the silica and surrounding environment, respectively.

If the injected light at the MFC input port 1 is unpolarized, the normalized power at the output port is described by:

(8) $$P=\frac{1}{2}\left[1+\cos\left(\left(C_{x}+C_{y}\right)\times L\right)\cos\left(\left(C_{x}-C_{y}\right)\times L\right)\right]$$

where *L* is the coupling region length of the MFC.

MFCs are relatively simple passive components which, due to their high performance and low cost, are widely exploited in telecommunications for power splitters, interferometers, and coarse wavelength division multiplexing. In the last decade, optical fiber couplers have been also applied to sensing applications such as pressure [44], micro fluidic flow meter [45], force [46], magnetic field [47], ultrasound [48,49] and temperature [50,51]. 

Due to the MFC small size, its transmission and coupling characteristics are affected by the external medium because of evanescent wave coupling. The modal optical paths depend on the coupling region length, fiber diameter at coupler waist and supermodes effective indices, which in turn are affected by the refractive index of the surrounding medium and by the temperature. The environmental temperature affects the MFC refractive index, the fiber diameter, the fiber length, and also the ambient refractive index through the thermo-optic effect and thermal expansion. 

Thermometry is one of the areas where fiber couplers could be particularly useful and has been investigated for applications in the oil and gas industries, in electric circuits and structural health monitoring. Recently, MFCs have been investigated for thermal sensing because of their compactness, quick manufacturing, high sensitivity, and fast response time [50,51,52]. 

A compact temperature sensor based on MFC for low temperature measurement was first demonstrated in 2008 [51], where the MFC fabricated by flame brushing technique was coated with an organic-inorganic sol-gel film. Because of the organic dopants among the network of silica material, the sol-gel has a higher thermo-optic coefficient than pure silica, and has been used to measure temperatures in the range −50 °C to 100 °C with a temperature sensitivity of *S* ~ 0.17 nm/°C. In 2008, a MFC waist region [53] was inserted in a thermo-optic external medium, water and temperature sensitive glycerin mixture. In order to measure the temperature sensitivity, the devices were packaged into a steel tube after inserting them into an external medium. By optimizing the external liquid refractive index (*n_ex_* = 1.440) at room temperature, the MFC sensor exhibited a sensitivity of *S* ~ −1.5 nm/°C in the temperature range *T* ~ 25 °C to 60 °C. In 2013 a fused 4 μm waist diameter MFC integrated with liquid crystals (LC) [54] was partially embedded in a low refractive index UV curable polymer (Efiron PC-363) to increase the robustness of the sensor and revealed a temperature sensitivity of *S* ~ 0.5 nm/°C. To enhance the sensitivity, the LC material was coated above the exposed central part of the MFC waist. The temperature measurement was performed in temperature range from 20–70 °C to ensure low viscosity of the LC. A temperature sensitivity of −0.7 nm/°C was achieved when the LC was coated on the coupler waist. 

MFC thermometry for monitoring the temperature of seawater has been presented in 2015 [55] by coating MFC with polyimide (PI). The MFC was assumed to be a weakly fused coupler with a 1.4 μm waist region and a 50 nm thick PI coating. A temperature sensitivity of 1.17 nm/°C was achieved as the seawater temperature was varied from 0 to 35 °C. High sensitivity temperature sensing in seawater [56] was achieved when 1% ethyl cellulose ethoce (EC) solvent was coated on the MFC uniform waist to strengthen the device, showing that the temperature sensitivity in seawater can be varied with the seawater salinity. At salinity levels of 38%, *S* ~ −1.13 nm/°C for seawater temperatures in the range 17 °C to 31.6 °C. 

The highest sensitivity of temperature sensors based on MFC coated or covered with thermo-optic materials has been reported in 2018 [57], when a fused MFC was inserted in a capillary tube and filled with refractive index matching liquid: both packaged capillary material and infilled liquid influenced the temperature sensitivity. The sensitivity of the sensor for different injected liquids was demonstrated by measuring the wavelength shift of two MFCs which are infilled with refractive index matching liquid and anhydrous ethanol when the temperature was varied in the range 35–45 °C. The temperature sensor based index matching liquid infilled MFC reveals higher sensitivity than the ethanol infill. The influence of the capillary material on the sensitivity was also demonstrated by encapsulated the MFC with the same waist diameter in Teflon and silica capillary tubes. The refractive index matching liquid was injected into both Teflon and silica tubes resulting in the highest temperature sensitivity of *S* ~ 5.3 nm/°C for the Teflon capillary tube.

**Figure 12:**
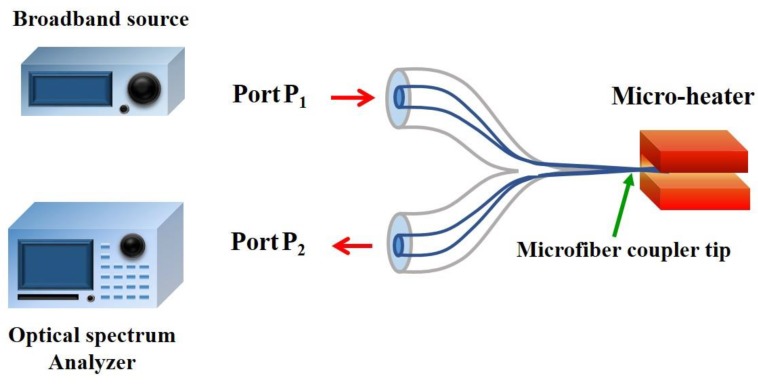
Experimental setup for temperature measurement using a bi-conical MF coupler tip (MFCT).

To measure temperatures higher than 200 °C, the use of pure silica MFC was investigated in 2012 [58,59]. The effect of MFC waist diameter on the temperature sensitivity was monitored from the wavelength shift within the temperature range 701 °C to 1029 °C, showing a temperature sensitivity of 31.1 pm/°C for a ~2.5 μm MFC waist diameter. In the same year, a large temperature range from ~247 °C to ~1283 °C [50,60,61] was measured using a MFC tip. The MFC was fabricated by using the ceramic micro-heater and it was cut at the center of coupler waist to create the MFC tip. In the temperature measurement, the same micro-heater used to fabricate the MFC was used to applied heat to the sensor (Figure 12). This sensor exhibited a temperature sensitivity of *S* ~ 11.96 pm/°C and a fast response time of 16.6 ms. 

**Figure 13:**
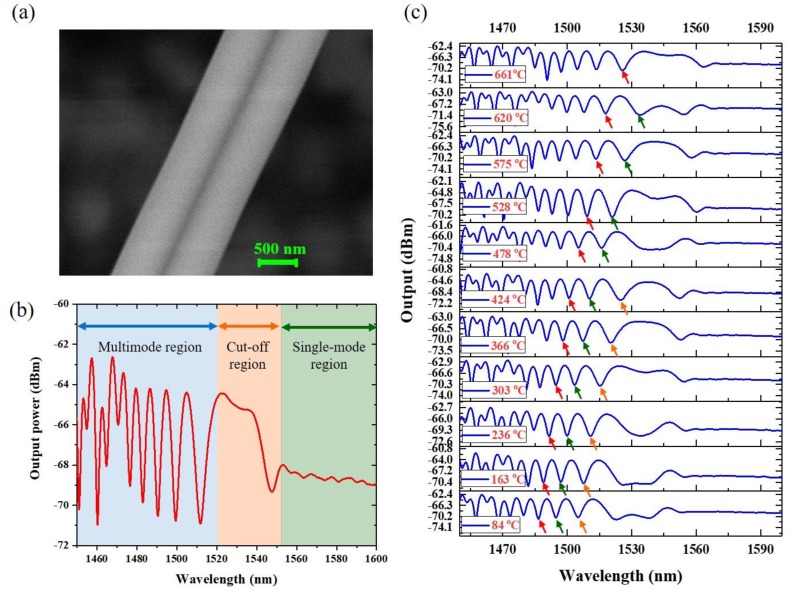
(**a**) SEM image of the MFC waist region. (**b**) MFC output power spectrum. (**c**) MFC transmission spectrum at different applied temperatures.

An ultra-compact temperature sensor based on pure silica MFC operating near its cut-off region has been demonstrated in 2017 [62] and relied on the interference patterns of the supermodes approaching the higher order modes cut-off region of the coupler. Although the coupler supports multiple supermodes, only the first two are excited, resulting in the single mode operation at its fiber pigtails. When the diameter (a) of a single nanofiber at the coupler waist is smaller than 1 micron, only one supermode is supported (Figure 13a). In the multimode region, the beating between two supermodes gives oscillations with high extinction ratios. In the cut-off region, the two supermodes stop beating as one of the supermodes is weakly guided due to the small diameter of the coupler and therefore the two supermodes have a very large difference between their propagation constants β_even_ ≠ β_odd_. Furthermore, since the odd supermode has a much larger fraction of its power in the evanescent field, it is excited less efficiently than the even supermode. For this reason, the power is not distributed equally between the even and odd supermodes and so full power exchange during beating is not possible, leading to a very small dip at cut-off region in Figure 13b and a relatively flat transmissivity over nearly 40 nm. At longer wavelengths, only one supermode is supported by the nanofiber coupler (NFC), resulting in a constant output at both ports, as shown in the single-mode region in Figure 13b. In this work, three wavelength dips close to the cut-off region were monitored with the changing of temperature. The wavelength dips in the coupler output arise from the destructive inference of the two beating supermodes. Because of the thermo-optic effect, any temperature increase causes an increase of the fiber coupler refractive index, thus of the supermodes effective refractive index. This change causes the destructive interference to shift to longer wavelengths and the weak guidance of the odd supermode at the cut-off region results in the disappearance of two wavelength dips close to the cut-off region when the temperature is higher than 620 °C (Figure 13a–c). Since the thermo-optic coefficient is positive, the refractive index increases with temperature, thus the sensitivity of dips that survive at the highest temperature will be the highest. Additionally, at wavelengths close to cut-off, the odd supermode extends considerably out of the silica, resulting in a stronger change in the effective refractive index difference of the supermodes. By measuring the temperature using the pure silica NFC operating near cut-off region, the highest temperature sensitivity of 60 pm/°C can be achieved.

### 5.3. Grating-Based Micro/Nanofibers

Optical fiber gratings are periodic perturbations of the fiber core/cladding refractive index. Like optical fiber gratings, MF gratings (MFG) have attracted increasing interest [63]. Thanks to their strong near-field interaction with surrounding materials, MFGs offer special advantages for temperature sensing including low detection limit, large dynamic range, and fast response. According to the grating period, the MFGs are categorized into two types which are MF Bragg grating (MFBG) and MF long period grating (LPG).

#### 5.3.1. Microfiber Bragg Gratings 

In principle, a MFBG is a miniaturized copy of a standard fiber Bragg grating (FBG) [63,64,65,66,67]. However, as it often exploits the refractive index difference between MF and surrounding environment, the index-contrast of the MFBG is usually much higher, resulting in a much shorter length. 

Temperature affects the Bragg wavelength shift through the thermo-optical effect and thermal expansion. The temperature sensitivity of sensors based MFBG can be defined as:

(9) ST=dλBdT=2Λ(σ∂neff∂nfT+rαT∂neff∂r+neffαT)

where *σ_T_* (1.2 × 10^−5^/°C and *α_T_* (5.5 × 10^−7^/°C) are the thermo-optic coefficient and the thermal expansion coefficient of fused silica, respectively. 

A temperature sensor based on the power reflected by MFBG has been reported in [68] using a 4.69 cm long MFBG inscribed with a frequency doubled argon laser through a uniform pitch phase mask. The MFGB was encapsulated in a metallic shield with a high thermo-optic coefficient and the reflected power from two MFBGs were monitored in the temperature range from 24 °C to 42 °C. 

**Figure 14:**
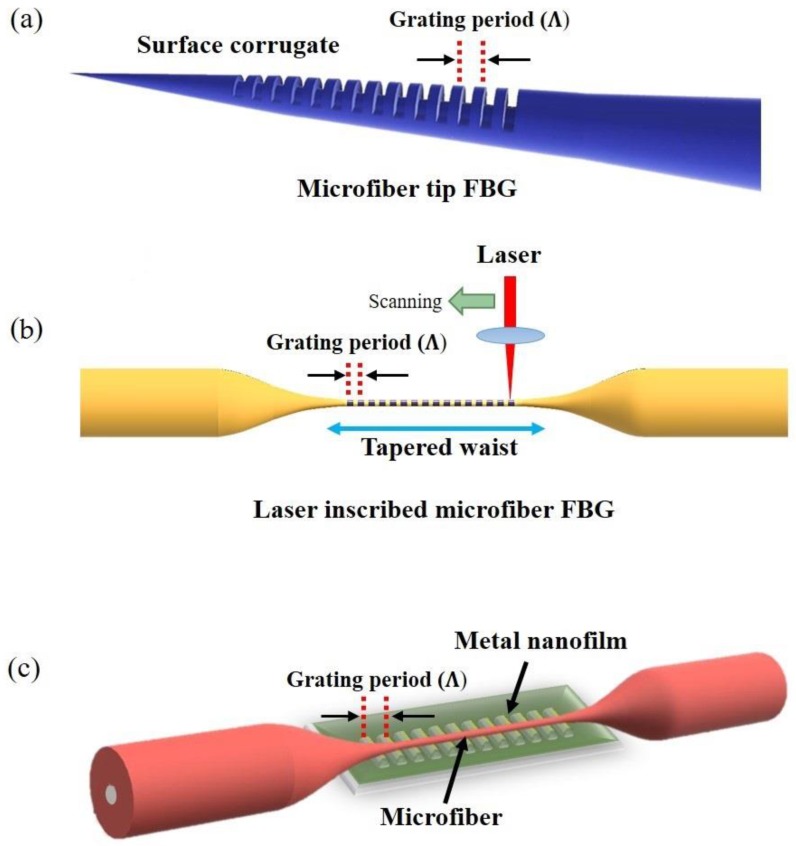
(**a**) Schematic of the FIB inscribed grating at the MF tapered tip for temperature sensor; (**b**) The schematic of laser inscribed Bragg grating in MF; (**c**) The schematic of a MF placed on top of metal nanofilm Bragg grating.

In 2011, a compact temperature sensor based on first order Bragg grating in a MF transition region was manufactured using focused ion beam (FIB) milling [69,70]. The 61 periodic grooves were inscribed on the surface of the 6.5 μm diameter tapered MF with a grating period of 600 nm (Figure 14a). The Bragg wavelength shift was measured in the temperature range 21 °C to 440 °C with sensitivity of 20 pm/°C. A multiplexed optical temperature sensing system using MFs and NFs with Bragg gratings as end reflectors was proposed in 2012 [71] and provided a sensitivity of 9.7 pm/°C between 20–70 °C. A 12 μm long device comprising an 11-period FBG engraved in a 5 μm diameter MF tip by focused ion beam milling as revealed in Figure 14b [72] exhibited a temperature sensitivity of 22 pm/°C. A high sensitivity MFBG sensor based presented in 2016 [73] relied on a 3.75 μm waist diameter MF connected to standard single SMF using a fusion splicer. The Bragg grating was inscribed using the femtosecond laser via the point by point technique. The MF FBGs were then molded with acetal and Sn-Ag-Cu alloy that have much higher coefficients of thermal expansion than pure silica. This sensor shows the highest sensitivity of 479.48 pm/°C in the temperature range 60 °C to 95 °C. 

#### 5.3.2. Microfiber Long Period Grating 

Light transmission in waveguides with periodic imperfections [74] results in mode conversion/coupling and was studied in both rectangular and round waveguides [75]. The LPG was first proposed in 1996 [76] and essentially introduces a coupling between the fundamental forward-propagating core mode and forward-propagating cladding mode. The LPG resonance wavelength is defined by the equation below [77]:

(10) $$\lambda =\left[n_{eff}\left(\lambda \right)-n_{clad}^{i}\left(\lambda \right)\right]\Lambda$$

where Λ is the period of the LPG, and $n_{eff}\left(\lambda \right)$ and $n_{clad}^{i}\left(\lambda \right)$ represent the effective refractive indices of the fundamental guided mode and the cladding mode of order *i*, respectively.

**Figure 15:**
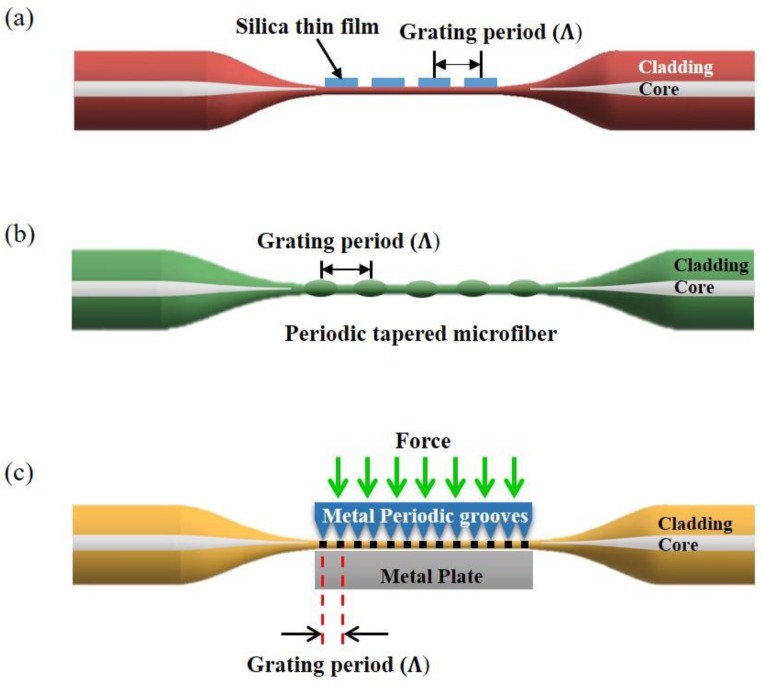
(**a**) Silica thin film long period grating on MF; (**b**) Schematic of MF LPG based on periodic tapered waist; (**c**) Schematic diagram of mechanical induced MF LPG.

LPG inscribed in a straight MF undergoes a spectral shift in response to a change in the ambient conditions and have been reported for optical sensing [78]. A temperature sensor based on MF LPG has been reported in 2010 [79] by coating a thin silica film with a period of 200 μm on a 30 μm diameter tapered MF (Figure 15a). The LPG period increases for increasing temperature resulting in the shift of resonance wavelength at the output. By optimizing the grating period and length of grating, an extremely high temperature sensitivity of *S* = 62.9 nm/°C was achieved. A thermometer based on a MF LPG was fabricated by periodically tapering a 76 μm diameter MF with a grating period of 1.3 mm as shown in Figure 15b [80]. This sensor simultaneously measured strain and temperature with the temperature sensitivity of *S* ~ 49.6 pm/°C and strain sensitivity of *S* ~ −0.55 nm/mε. Functionalization can improve the sensor sensitivity [81]: a 25-points 100 μm period MF LPG was inscribed with a UV laser on top of a PMMA coated 5.4 μm diameter MF and was used to measure temperature from 20 °C to 50 °C providing *S* ~ 385.11 pm/°C. LPG can be also induced mechanically in MF [82]. A 5-cm long aluminum plate with 500 μm-period and 300 μm grooves was placed under a 60 μm MF (Figure 15c): by applying heat to the periodic groove plate, this structure exhibited *S* ~ 21 pm/°C at *T* ~ 400 °C. 

### 5.4. Interferometer-Based Micro/Nanofibers

Optical interferometers are among the most powerful tools for optical sensing, as they transform a change in length or refractive index in a phase/intensity change that can be measured with very high precision. As miniaturized optical interferometers, the MF Mach-Zehnder interferometer (MZI) and Fabry-Perot interferometer (FPI) are of interest for phase-sensitive optical measurement with compact size and high sensitivity. 

**Figure 16:**
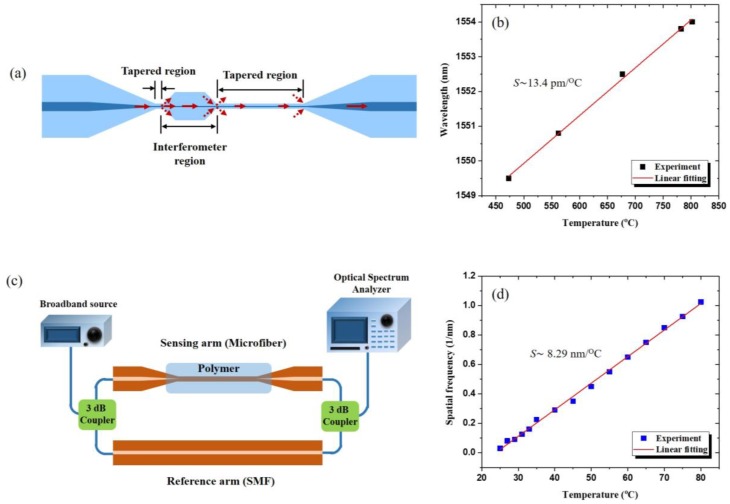
(**a**) Schematic diagram of the fabricated inline MF MZI; (**b**) Shift in the transmission wavelength dip of the inline MF-MZI for temperature increases; (**c**) Experimental scheme for the polymer coated MF MZI; (**d**) Spatial frequency shift (∆*f*) of the polymer overlay MF-MZI for increasing temperatures.

In 2009, temperature and refractive index measurement [83] were performed using a MF-MZI formed by tapering two sections in the same fiber at the distance of 525 μm. Measured temperature sensitivity of *S* ~ 0.077 nm/°C for the interference order of 144 was exhibited. In 2010 a MF tip Fabry-Perot modal interferometer (FPMI) [84] was created at the tip of <10 μm diameter MF coated by a thin layer of aluminum, by FIB milling a 4.4 μm wide microcavity. Temperatures from room temperature to 520 °C with a sensitivity of *S* ~ 20 pm/°C near *λ* ~ 1550 nm was obtained. High temperature thermometry based on inline MF-MZI [85] was demonstrated creating an interferometer from two tapered regions by using the flame-brushing technique (Figure 16a). This sensor was used to measure temperatures up to 800 °C and it revealed a temperature sensitivity of *S* ~ 13.4 pm/°C. A compact and high temperature sensor-based MF-MZI was presented in 2017, based on waist-enlarged fiber bitapers [86]. The MZI was composed of a thin core fiber (TCF) sandwiched between two SMFs with two waist-enlarged bi-tapers and allowed to measure temperatures of 1000 °C with *S* ~ 0.087 nm/°C. 

A low refractive index UV curable polymer was used to clad a 10 μm diameter MF to generate the sensing arm of MZI [87] (Figure 16b), providing *S* ~ −8.29 nm/°C. 

## 6. Resonant-Based Micro/Nanofiber Temperature Sensors

MF resonant sensors exploit the increased optical path length experienced by light when propagating at the resonance wavelength in MF resonators. The environment temperature change has effects on the MF length, diameter, and refractive index variation, which will be translated as change in the resonant frequency of the resonator.

Microresonators (MR) are submicron devices exploiting the MF feature of light self-coupling through its large evanescent field [88]. MF resonators are manufactured by coiling a MF onto itself, which will cause the modes propagating in adjacent sections to overlap and couple [64]. Resonators are often classified by *FSR*, *Q*-factor and Finesse, as explained hereafter.

*FSR* is the free spectral range and it represents the wavelength/frequency difference between two successive minima/maxima in the resonator transmission:

(11) $$FSR=\frac{\lambda^{2}}{n_{eff}L}$$

where $n_{eff}$ is the effective index of a mode, k is the wave number k = 2π/λ, β is the propagation constant and L is the cavity length.

*Q*-factor: The quality factor *Q* of an oscillating system is a measure of the intensity of the dumping of its oscillation and it can be represented as the ratio of the energy stored with respect to the energy dissipated per cycle. It can be written as:

(12) $$Q=\frac{\lambda_{r}}{FWHM}$$

where $\lambda_{r}$ is the resonance wavelength, *FWHM* is the resonance Full-width at half-maximum (linewidth).

Finesse (*F*) is the ratio between the *FWHM* and the *FSR* and it depends on the diameter of the coil:

(13) $$F=\frac{FSR}{FWHM}$$

The major MF microresonators have been in the form of loop [89], knot [90], and coil [91].

### 6.1. Loop Resonator (LR)

#### 6.1.1. Manufacture and Properties

**Figure 17:**
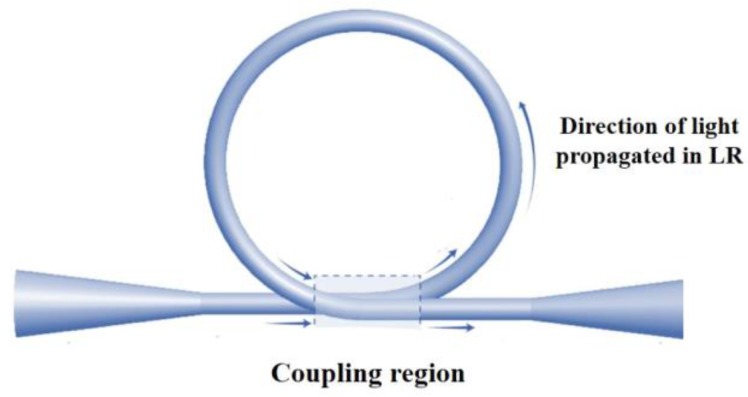
Schematic of loop resonator (LR) while arrows represent the direction of light propagated in LR.

This resonator is the simplest form of MF MR. Its fabrication simply includes bending a MF into a self-coupling loop [92] and its dimensions are adjusted by careful adjusting the position of the sections adjacent to the loop. Figure 17 shows a schematic of the LR with the coupling region. This resonator has the highest reported *Q* factor, in excess of 10^5^. The LR drawback is that its shape and coupling is maintained by electrostatic and van der Waals forces between the coil different parts of the coil, and it has a limited stability that is strongly related to environmental conditions [89,93]; embedding can be a solution to temporal stability, yet it can induce considerable variations of the output of the device. Fusing the loop contact point with CO_2_ laser [94] was proposed as a long term solution, but it hinders the LR *Q* factor.

#### 6.1.2. LR Temperature Sensor

A LR temperature sensor [89] was first demonstrated in 2006, in the form of freestanding loop and it allowed for the measurement of temperature variations of 0.4 °C with a response time *t* ~ 3 ms. 

The thermal response of LR was used as a tool to tune the resonance wavelength, and a LR wrapped on a copper wire provided a linear dependence of wavelength shift on the electric current with a slope of ~26.5 pm/A [94]. 

The use of the resonance extinction ratio for thermometry has also been studied in embedded loop resonators [95], an provided a sensitivity of *S* ~ 0.043 dB/°C. 

A MLR integrated in a Sagnac loop reflector provided a linear response to both temperature and refractive index variations with a temperature sensitivity of *S*_T_ ~ 20.6 pm/°C in the temperature range of 30–130 °C. This approach combines the physical strength of Teflon coating with the high responsivity of the MF [96].

### 6.2. Knot Resonator (KR)

#### 6.2.1. Manufacture and Properties

**Figure 18:**
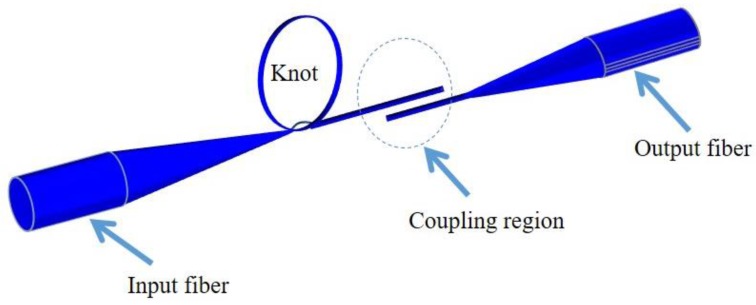
Schematic of the original methodology used for manufacturing KR.

The KR is a MF resonator manually manufactured from MFs. This resonator shows good stability, Q factors up ~57,000 and finesses as large as 22. The first KR (Figure 18) was manufactured by micromanipulation under an optical microscope. Initially a loop was formed from the freestanding end of the single ended MF, which is tightened to a smaller knot by pulling the free end of the fiber. Another MF was required to evanescently collect light from the knot [90,97].

**Figure 19:**
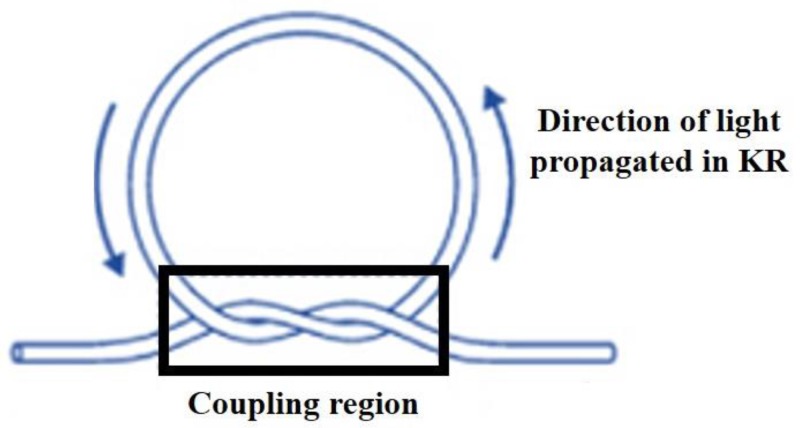
Modified technique to manufacture the KR.

A simplified technique (Figure 19) to fabricate the KR [98], suggests manufacturing the KR from a double-ended tapered fiber. In this technique light is launched and collected in the same MF forming the knot, which enhances the KR stability. The fabrication process involves knotting the untapered end of the MF and then decreasing the knot diameter by pulling the fiber ends. This technique has produced a low loss KR with high finesse of 104 and a Q factor of ~10^4^.

#### 6.2.2. KR Temperature Sensors

KRs have been demonstrated for temperature sensing [99]. In one of the early demonstrations, silica and polymer resonators were placed between two MgF_2_ crystal plates, resulting in an induced resonance wavelength shift of *S*_T_ ~ 52 pm/°C in the range 30–700 °C with a response time of *τ* ~ 1 ms for the silica sensor and *S*_T_ ~ 266 pm/°C, operation range of *T* ~ 20–80 °C, and a *τ* ~ 5 ms for the PMMA (poly-methyl-methacrylate) sensor.

KRs temperature sensors embedded in low refractive index fluoropolymers (Efiron UV PC-373) were demonstrated and provided high sensitivity (*S* ~ 270 pm/°C) in the temperature range *T* ~ 25–140 °C [100]. Cascaded KRs were used to demonstrate high-precision and simultaneous multi-point temperature sensing [101]. A seawater temperature sensor based on a KR was demonstrated in [102]. Results show that sensing sensitivity increases with the increasing MF diameter from 2.30 μm to 3.91 μm, which was attributed to the larger overlap with seawater associated with MFs with smaller diameters. The evanescent field and the influence of the negative thermo-optic coefficient of seawater weaken at increasing fiber diameters, and results in decreased sensitivity. In addition, sensitivity also increases with increasing probing wavelength because of increases in the negative thermos-optic coefficient of seawater [103].

A structure composed of a MF KR and an abrupt taper-based MZI was proposed in [104] using CO_2_ laser processing and showed a transmission spectrum with two different components having different sensitivities to different physical and chemical parameters. The sensor was characterized for temperature and refractive index. For temperature sensing in water, the MZI component presented *S* ~ −196 pm/°C while the KR component showed *S* ~ 25.1 pm/°C, for T variations of 12 °C.

### 6.3. Microcoil Resonator (MR)

#### 6.3.1. Manufacture and Properties

Microcoil resonator is a tridimensional (3D) all-fiber resonator based on continuous self-coupling between adjacent MF turns. It was first theoretically proposed [91] in 2004, and implemented experimentally in 2007 [88]. Although theoretical Q factors of 10^9^ were predicted, competing with those achieved using whispering-gallery mode resonators, the highest Q factor experimentally achieved in an MR was 220,000 [105].

**Figure 20:**
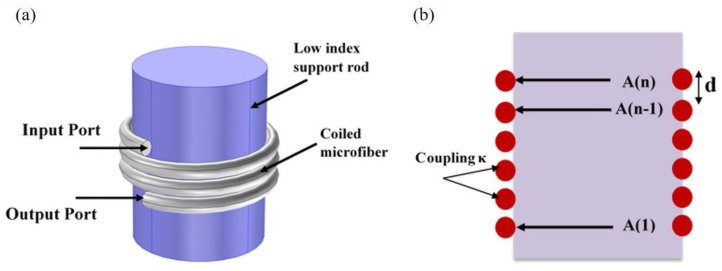
Microcoil resonator (**a**) Schematic of the resonator: a MF coiled around a solid cylindrical rod; (**b**) Cross section.

These resonators are fabricated by coiling a MF around a support rod which has a lower refractive index than the MF (Figure 20). Unlike LR and KR, it provides self-coupling along the whole coil between adjacent turns, and light can be trapped inside the resonator and experience enhanced slow/fast light phenomena [106].

#### 6.3.2. MR Temperature Sensor

Although the MR resonator represents the best candidate for compact, efficient temperature sensing, the implementation of this geometry for temperature sensing has been limited. This is mainly due to two facts: the fabrication complexity of this resonator that requires a precise control of the speed and the angle of the rotation of the MF, and the MR packaging with a material suitable for temperature monitoring (embedding has often been performed with UV curable polymers (Efiron 373) [24] and with Teflon [22]) without affecting the Q factor. Temperature detection using a polymer coated MR was demonstrated [107,108] using a weakly coupled MR. In this work, a 2 μm-diameter silica MF was wrapped around a 1 mm-diameter Teflon rod to form a 3-turns MCR, and subsequently embedded in a UV curable polymer, providing a thermometer with *S* ~ 95 pm/°C up to ~80 °C. Another study [109] showed that MR can be tailored to be temperature insensitive by embedding the MR in Teflon with opposite thermo-optic coefficient. This behavior was explained by the temperature dependence of the MR MF size, which controls the ratio of thermal effect contributions from silica and polymer. By using a ~3 μm-diameter MF, the MCR temperature dependence was compensated to less than 6 pm/°C. 

## 7. Conclusions

A variety of temperature sensors based on micro/nanofibers have been proposed and experimentally demonstrated. Benefitting from their small sizes, excellent optical confinement, high-fractional evanescent fields, bend insensitivity, and possibility to be made into resonators, MFs based temperature sensors have shown special advantages over conventional optical fiber sensors, including a very fast response time.

In this review, MF temperature sensors have been arranged in groups based on the resonant and nonresonant nature of the sensor. Table 1 summarizes the results published on the subject. MFs-based temperature sensors have been investigated for both low and high temperature (>500 °C) measurements. To increase the sensor sensitivity, most of low temperature sensors are based on the MF coated or infilled with high thermo-optic coefficient materials. Until now, the presented low temperature sensors based on MFs reveals the maximum temperature sensitivity of 62.9 nm/°C (item 20 in Table 1). The pure silica based MFs structures have been used mostly to fabricate high temperature sensors owing to their high softening temperature. Although silica has a low thermo-optic coefficient, by optimizing the MF structure, thermometer based on pure silica have shown sensitivities of *S ~* 87 pm/°C (item 27 in Table 1) and a maximum measured temperature of 1283 °C (item 13 in Table 1).

## Figures and Tables

**Table 1:** Summary of MF Thermometers.

Structure	Sensitivity	Temperature	Reference
MF			
1. MF coated thermochromic film	0.043 dB/°C	10–40 °C	[29]
2. IPA filled MF	3.88 nm/°C	20–50 °C	[30]
3. Non-adiabatic MF packed with PDMS	3.102 nm/°C	20–48 °C	[31]
4. Metal coated MF	2.44 nm/°C	20–120 °C	[33]
5. Graphene assisted MF	0.1052 dB/°C	30–80 °C	[35]
MFC			
6. Sol-gel coated MFC	0.17 nm/°C	−50–100 °C	[51]
7. MFC filled with water + glycerin	1.5 nm/°C	25–60 °C	[53]
8. Liquid crystal coated MFC	0.7 nm/°C	20–70 °C	[54]
9. Polyimide coated MFC Seawater temp	1.17 nm/°C	0–35 °C	[55]
10. Ethyl cellulose ethoce(EC) coated MFC	1.13 nm/°C	17–31.6 °C	[56]
11. MFC infilled with RI matching liquid using Teflon tube	5.3 nm/°C	35–45 °C	[57]
12. Pure silica MFC	31.1 pm/°C	701–1029 °C	[59]
13. Pure silica MFC tip	11.96 pm/°C	247–1283 °C	[50]
14. Pure silica based NFC	60 pm/°C	84–661 °C	[62]
Gratings			
15. Encapsulated MFBG in metallic shield		24–42 °C	[68]
16. Micromachining FBG on pure silica MF tip	20 pm/°C	21–440 °C	[69]
17. Pure silica MFBG as end reflector	9.7 pm/°C	20–70 °C	[71]
18. MFBG at MF tip	22 pm/°C	23–228 °C	[72]
19. Concatenated SMF-MFBG_SMF coated with acetal	479.48 pm/°C	60–95 °C	[73]
20. Coated silica thin film on MF	62.9 nm/°C	24.5–30 °C	[79]
21. Periodic tapered MF LPG	49.6 pm/°C	30–180 °C	[80]
22. PMMA coated LPG on top of MF	385.11 pm/°C	20–50 °C	[81]
23. Mechanical induced MF LPG	21 pm/°C	400 °C	[82]
Interferometer			
24. MZI two fiber taper	0.077 nm/°C	20–60 °C	[83]
25. FPI based micro-notch on aluminum coated MF tip	20 pm/°C	19–520 °C	[84]
26. Two tapered formed MF MZI	13.4 pm/°C	800 °C	[85]
27. Thin core fiber combine with waist enlarge fiber for fabricating microfiber MZI	0.087 nm/°C	1000 °C	[86]
28. UV coated MF for fabricate MZI arm	8.29 nm/°C	25 °C	[87]
Loop			
29. Loop resonator	26.5 pm/A		[95]
30. Resonance extinction ratio of LR	0.043 dB/°C		[96]
31. Sagnac loop interferometer	20.6 pm/°C	30–130 °C	[97]
Knot			
32. Silica nnot resonator placed between two MgF_2_ plates	52 pm/°C	30–700 °C	[100]
33. PMMA Knot resonator placed between two MgF_2_ plates	266 pm/°C	20–80 °C	[100]
34. Embedded KR in fluoro-polymer	270 pm/°C	25–140 °C	[101]
35. MKR for water temperature measurement	25.1 pm/°C	38–50 °C	[105]
MCR			
36. UV cure polymer coated on MCR coiled around teflon	95 pm/°C	80 °C	[109]
